# Erythropoietin Alleviates Burn-induced Muscle Wasting

**DOI:** 10.7150/ijms.38590

**Published:** 2020-01-01

**Authors:** Sheng-Hua Wu, I-Cheng Lu, Ming-Hong Tai, Chee-Yin Chai, Aij-Lie Kwan, Shu-Hung Huang

**Affiliations:** 1 Department of Anesthesiology, Kaohsiung Medical University Hospital, Kaohsiung Medical University, Kaohsiung, Taiwan.; 2 Department of Anesthesiology, School of Medicine, College of Medicine, Kaohsiung Medical University, Kaohsiung, Taiwan.; 3 Department of Anesthesiology, Kaohsiung Municipal Hsiao-Kang Hospital, Kaohsiung Medical University, Kaohsiung, Taiwan.; 4 Department of Anesthesiology, Kaohsiung Municipal Ta-Tung Hospital, Kaohsiung, Taiwan.; 5 Center for Neuroscience, National Sun Yat-Sen University, Kaohsiung, Taiwan.; 6 Department of Biotechnology, Southern Taiwan University of Science and Technology, Tainan, Taiwan.; 7 Departments of Pathology, College of Medicine, Kaohsiung Medical University, Kaohsiung, Taiwan.; 8 Faculty of Medicine, College of Medicine, Kaohsiung Medical University, Kaohsiung, Taiwan.; 9 Institute of Biomedical Sciences, National Sun Yat-Sen University, Kaohsiung, Taiwan.; 10 Department of Surgery, School of Medicine, College of Medicine, Kaohsiung Medical University, Kaohsiung, Taiwan.; 11 Division of Plastic Surgery, Department of Surgery, Kaohsiung Medical University Hospital, Kaohsiung, Taiwan.; 12 Regeneration Medicine and Cell Therapy Research Center, Kaohsiung Medical University, Kaohsiung 807, Taiwan.

**Keywords:** Erythropoietin, Muscle fiber atrophy, Burn injury, Apoptosis Inducing Factor, Transforming Growth Factor beta1

## Abstract

**Background:** Burn injury induces long-term skeletal muscle pathology. We hypothesized EPO could attenuate burn-induced muscle fiber atrophy.

**Methods:** Rats were allocated into four groups: a sham burn group, an untreated burn group subjected to third degree hind paw burn, and two burn groups treated with weekly or daily EPO for four weeks. Gastrocnemius muscle was analyzed at four weeks post-burn.

**Results:** EPO attenuated the reduction of mean myofiber cross-sectional area post-burn and the level of the protective effect was no significant difference between two EPO-treated groups (p=0.784). Furthermore, EPO decreased the expression of atrophy-related ubiquitin ligase, atrogin-1, which was up-regulated in response to burn. Compared to untreated burn rats, those receiving weekly or daily EPO groups had less cell apoptosis by TUNEL assay. EPO decreased the expression of cleaved caspase 3 (key factor in the caspase-dependent pathway) and apoptosis-inducing factor (implicated in the caspase-independent pathway) after burn. Furthermore, EPO alleviated connective tissue overproduction following burn via transforming growth factor beta 1-Smad2/3 pathway. Daily EPO group caused significant erythrocytosis compared with untreated burn group but not weekly EPO group.

**Conclusion:** EPO therapy attenuated skeletal muscle apoptosis and fibrosis at four weeks post-burn. Weekly EPO may be a safe and effective option in muscle wasting post-burn.

## Introduction

Muscle fiber atrophy is a hallmark of several critical disorders, including burn injury 1-4. A decline of skeletal muscle impairs patient recovery, including prolonged mechanical ventilation use, poor wound healing, and increased risk of infection 5-7. Burn injury is considered the most devastating injury that may cause long-term muscle wasting and a decrease in muscle strength over several months 8-10. Persistent muscle atrophy following burn impedes full recovery 9, 11. The underlying cellular mechanisms leading to burn-induced muscle wasting remain elusive and effective therapeutic options are in demand for these patients. A consequence of central nerve system denervation following burn injury subsequently causes muscle wasting. A burn mice study indicated that upregulation of cytokines and chemokines post-burn resulted in microglia activation, motor neuron degeneration and muscle loss 12. In addition, several possible pathogeneses within skeletal muscle contribute to burn-induced muscle wasting. Recent studies showed the level of muscle pro-catabolic or muscle-specific secretory factor was increased under burn serum stimulation 13, 14. Persist hypermetabolic state in response to burn-induced proinflammatory cascades and catabolic hormones ultimately caused skeletal muscle breakdown 8, 15, 16. The activation of skeletal muscle cell autophagy also played a role in skeletal muscle wasting following burn 17. A possible bone secreted factor, TGF-β, also involved in burn-induced muscle cachexia by increasing oxidative damage to muscle 18.

Furthermore, previous studies have proposed skeletal muscle cell apoptosis was increased following burn and involved in classical caspase-dependent pathways 19-23. As Yasuhara et al reported increased apoptosis and caspase-3 activity in skeletal muscle within a few hours after burn in a rat model 20. Other burn model showed maximal apoptosis occurred on four days after injury and caspase-3, -8 and -9 activity increased in tibialis anterior muscle 22. Moreover, the role of caspase-independent mediated apoptosis has rarely been discussed in burn injury models. The release of apoptosis-inducing factor (AIF) might associate with an increased skeletal muscle apoptotic potential, and result in muscle atrophy 24. Muscle biopsies of rat and human suggested age-related muscle loss might be involved in the activation of AIF 25, 26. Another process impairs muscle regeneration associated with overproduction of extracellular matrix (ECM) 27. Abnormal muscle repair and excessive ECM deposition consequently progress toward fibrosis post-acute phase of trauma, which often causes poor response to pharmaceutical therapy. Burn injury often results in hypermetabolic state and production of various inflammatory factors 7, 28-31. Transforming growth factor beta 1 (TGF-β1) is a crucial factor to regulate ECM remodeling 32, 33 and drives tissue to fibrosis in chronic inflammatory diseases. Inhibition of TGF-β1 activity enhances tissue repair 34, 35. Research of burn scars reports that TGF-β1 acts through the Smad protein system to activate genes related to fibrosis 36, 37. In addition, a downstream effector of TGF-β1, connective tissue growth factor (CTGF) was sustained increase in several fibrotic conditions 37-41 included burn scars to regulate ECM synthesis. However, scanty data investigated their pro-fibrotic role in skeletal muscle at the post-acute phase of burn and possible therapeutic agents.

Erythropoietin (EPO) is a pleiotropic hormone whose primary function is to stimulate erythropoiesis. Its target receptors are expressed in several cell types including skeletal muscle 42-49. EPO has a tissue protective potential, including anti-inflammation, anti-apoptosis, and improving metabolic alteration 48, 50-53. EPO reduced cells apoptosis and inhibited pro-inflammatory cytokines in sepsis-induced lung injury model 54 as well as in kidney ischemia/reperfusion injury model 55. Neuroprotection of EPO was supported in ameliorating PC 12 cells against oxidative stress 56. EPO protected heart from fibrosis by suppressing TGF-β1, collagen and pro-inflammatory cytokines expression in rat cardiac fibroblasts and in a rat model of cardiac remodeling 57, 58. For critical trauma patients, EPO therapy might reduce mortality and improve outcome without increasing adverse events 59-61. However, the precise role and regulatory mechanisms of EPO in skeletal muscle remains uncertain 62. Human muscle biopsies showed EPO induces myogenic differentiation factor expression in satellite cells, which participate in muscle regeneration following 10 weeks EPO treatment 63 and improves type I muscle fiber diameter in hemodialysis patients 64. A diabetic mice model reported that 4 and 8 weeks EPO therapy reduced skeletal muscle insulin resistance by increasing autophagy and reducing apoptosis 65. EPO increased erythropoietin receptor expression and induced cell proliferation in C2C12 myoblasts and satellite cells 66. In addition, exercise training and erythropoietin attenuated muscle alterations in cancer cachexia 67. These data suggest the potential therapeutic role of EPO in muscle wasting post-burn.

In this rat study, we investigated the impact of burn on gastrocnemius muscle at four weeks post-burn and hypothesized EPO could prevent muscle wasting through anti-apoptosis and anti-fibrosis. We induced a burn trauma in the right hindpaw among three groups of rats, two treated with EPO, one group treated daily and the other group treated weekly. The pharmacological effect of EPO on caspase-dependent and caspase-independent mediated cell apoptosis was investigated. In addition, the anti-fibrogenic mechanism of EPO on TGF-β1-induced CTGF expression to regulated ECM synthesis was also investigated.

## Materials and Methods

The study used 24 adult, male Sprague-Dawley rats. The procedures were approved and conduced in accordance with the guidance of the Institutional Animal Care and Use Committee at Kaohsiung Medical University (IACUC Approval Number: 106047). On day 0 (D0), all animals received either a third-degree burn or sham burn injury and wound care following a previous thermal model 68. The rats were randomly allocated into four groups of six rats each: (1) a sham-control group (sham burn group) which received a sham burn and no drug treatment; (2) an untreated burn-only group (burn group); (3) a burn group treated with weekly EPO for four weeks (weekly EPO group) (5000 IU/kg i.p. at day 0, week 1, 2, 3 [D0, W1, 2, 3]); and (4) a burn group treated daily with EPO for four weeks (daily EPO group) (3000 IU/kg/day i.p. at day 0 to day 27 [D0-D27]).

The right hind paw of each rat topped with 100-g weight (to maintain a standardized surface contact) was placed, plantar side down, on a temperature-controlled metal surface for 10 seconds 68. The metal surface was set at 25 ± 0.5°C for the sham burn group. It was set at 75 ± 0.5°C for the three study groups, resulting in a third-degree thermal injury. The weekly and daily EPO groups both received recombinant EPO (Epoetin, Recormon, Roche) administered intraperitoneally (i.p) immediately after the burn injury. Afterwards, two groups were treated with daily or weekly EPO as scheduled. Our preparation of EPO and the dosage we used were based on our previous study 69. Four weeks (W4) after burn, blood samples were collected to measure red blood cell (RBC) mass by an autoanalyzer (Bayer ADVIA 2120). Then all rats were euthanized by administration of Zoletil 50 overdose and perfused with 4% paraformaldehyde. The gastrocnemius muscle of the right limb of each rat was harvested for histological analysis.

Tissue samples from the mid-belly of the gastrocnemius muscle were fixed by formalin, embedded in paraffin and cut into 4-μm-thick sections. Serial tissue slices were mounted on glass slides, deparaffinized, and rehydrated in graded alcohol solutions. Muscle sections were stained with Hematoxylin and Eosin (H&E) staining (Abcam, Cambridge, MA) according to the manufacturer's directions and visualized by light microscopy. The average muscle cross-sectional area of each experimental group was acquired from six stained sections of each specimen using a Nikon eclipse E600 and capture with Nikon digital sight DS-5M. Images were obtained for the morphometric analyses with image analysis software IPP6.0 (Media Cybernetics, Bethesda, USA) after observing under a microscope.

To detect apoptotic cell death, TdT dUTP nick-end labeling (TUNEL) assay was performed according to the manufacturer's directions (Millipore, ApopTag fluorescein in situ apoptosis detection kit S7110). After incubated with 5% normal goat serum for 1 hour, the sections were incubated with cleaved caspase 3 (1: 200; Cell Signaling, Beverly, MA) or apoptosis-inducing factor (AIF, 1: 200, Abcam, Cambridge, MA) overnight at 4℃. Subsequently, all of them were incubated with Cy3-conjugated anti-mouse IgG secondary antibody (MerckMillipore, Bedford, MA) at room temperature for an additional 1 hour, rinsed 3 times with PBS for 5 minutes each, and mounted with a medium containing 4'6-diamidino-2phenylinodole (DAPI) for identifying nuclei. TUNEL index was calculated as the number of TUNEL-positive nuclei to the gross area of nuclei according to a previous study 25.

Muscular fibrotic changes were analyzed by immunofluorescence with fibronectin (1: 200, Novus Biologicals, Littleton, CO, USA), type I collagen (1: 200, Novus Biologicals, Littleton, CO, USA), type III collagen (1:200, Origene, Rockville, Maryland, USA) and phosphorylated Smad2/3 (1:200, Cell Signaling, Beverly, MA). The appropriate secondary antibody conjugated with goat anti-rabbit Cy3 (1:400, red, Millipore, Temecula, CA). All images were acquired using a fluorescence microscope (Leica DM 6000).

Western blotting of the tissue samples was performed as described previously 69. The antibodies used were atrogin-1 (1: 1000; affinity biosciences, Changzhou, China), cleaved caspase 3 (1: 1000; Cell Signaling, Beverly, MA), apoptosis-inducing factor (AIF) (1:1000, Abcam, Cambridge, MA), fibronectin (1:1000, Novus Biologicals, Littleton, CO, USA), type I collagen (1:1000, Novus Biologicals, Littleton, CO, USA), Type III collagen (1:1000, Origene, Rockville, Maryland, USA), transforming growth factor beta-1 (TGF-β1) (1:1000, Abcam, Cambridge, MA), connective tissue growth factor (CTGF) (1: 1000, Novus Biologicals, Littleton, CO, USA) and GADPH (1:2000, Sigma-Aldrich, Poole, Dorset, UK) were performed. The muscle lysates were centrifuged at 13,000× RPM at 4°C for 30 minutes. Each protein concentration of the supernatants was measured using bovine serum albumin as the standard. The above antibody-bound proteins were detected using chemiluminescence detection regents and the total signal was quantified using Image Lab.

All values were expressed as means ± standard error of the mean (SEM). All data were calculated according to the numerical data, as presented in the text. All statistical operations were performed using SPSS (ver.14.0, Chicago, IL, USA). A p < 0.05 was considered statistically significant. *p < 0.05. **p < 0.01.

## Results

### Weekly EPO did not cause significant erythrocytosis

To investigate whether two EPO regimens lead to the obvious erythrocytosis, we checked red blood cell (RBC) count of four groups (shown in Table 1). Compared to untreated burn rats, those treated with weekly EPO did not have significantly altered RBC count (7.84±0.99×10^6^ /uL vs. 8.07±0.88×10^6^/uL, p=0.069), while those treated daily EPO did (7.84±0.99×10^6^/uL vs. 10.41±0.87×10^6^/uL, p=0.001).

### EPO alleviated burn-induced muscle fiber atrophy and the expression of atrophy-related ubiquitin ligase, atrogin-1

**Figure 1:**
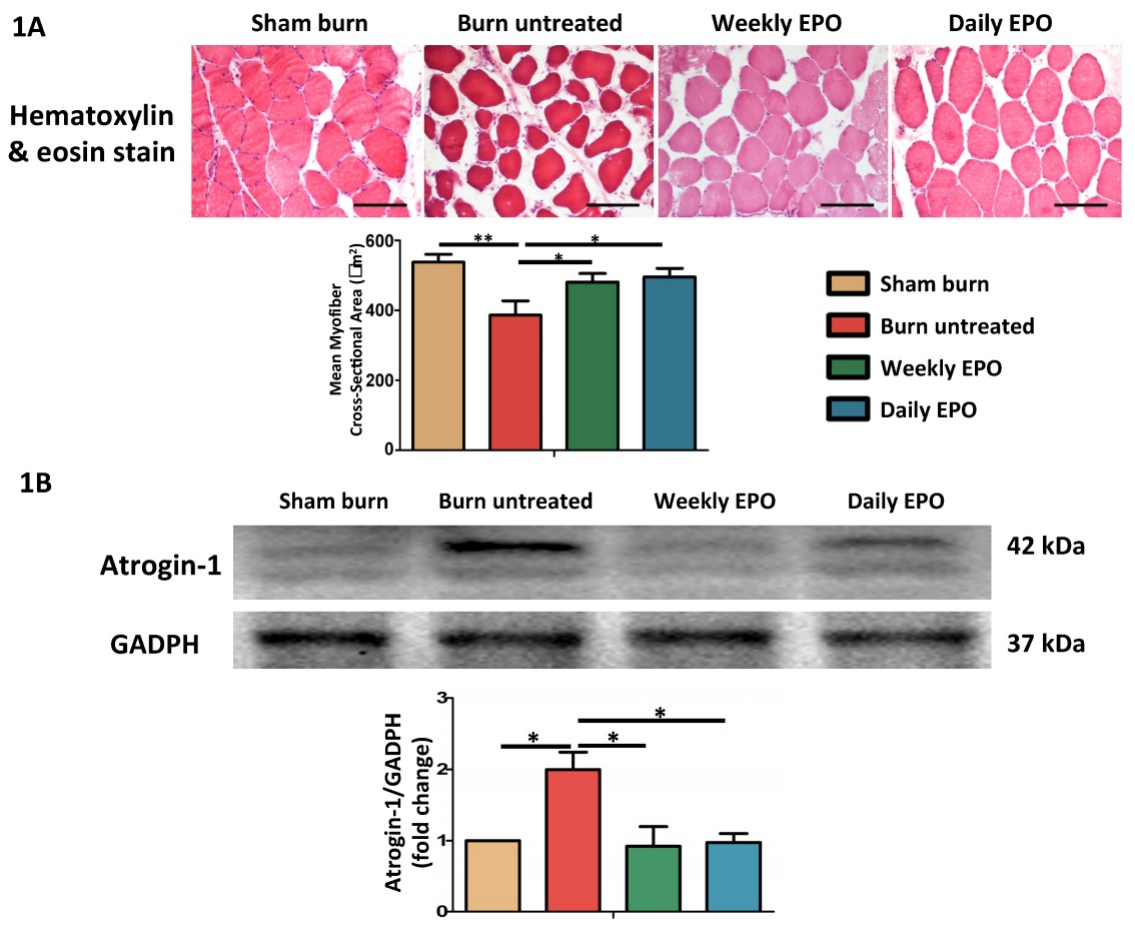
EPO on burn-induced muscle fiber atrophy and atrophy-related ubiquitin ligase, atrogin-1 (A) Representative H&E staining of gastrocnemius muscle section (200x). Average myofiber cross-sectional area of 4 groups. (B) Representative western blot of atrogin-1. EPO decreased the elevation of atrogin-1 post-burn. All error bars represented the SEM. *p<0.05, **p<0.01.

H&E staining showed significant muscle fiber atrophy at four weeks post-burn in Figure 1. Both EPO-treated groups attenuated the reduction in the mean cross-sectional area of myofibers. There was no significant difference between EPO-treated groups (p=0.784). Furthermore, atrogin-1 is an important regulator of ubiquitin-mediator protein degradation in skeletal muscle. An increase expression was found in aged rats with the decline of muscle mass 70 and during muscle atrophy 71. Our result showed that the atrophy-related ubiquitin ligase was significantly up-regulated in burn untreated group in comparison with sham groups (p=0.032). Compared to the untreated burn group, both EPO-treated groups attenuated the expression of atrogin-1 (Weekly EPO, p=0.048, Daily EPO, p=0.041).

### EPO attenuated burn-induced muscle apoptosis

In Table 2, compared to the sham burn group, the untreated burn group shows a significant increase in the number of TUNEL-positive nuclei (p=0.008). Compared to the untreated burn group, both EPO-treated groups had markedly decreased cell apoptosis (Weekly EPO, p=0.040, Daily EPO, p=0.028). In anti-apoptotic efficacy, weekly EPO was no inferior to daily EPO (p=0.08).

### EPO attenuates apoptosis post-burn by decreasing cleaved caspase 3

**Figure 2:**
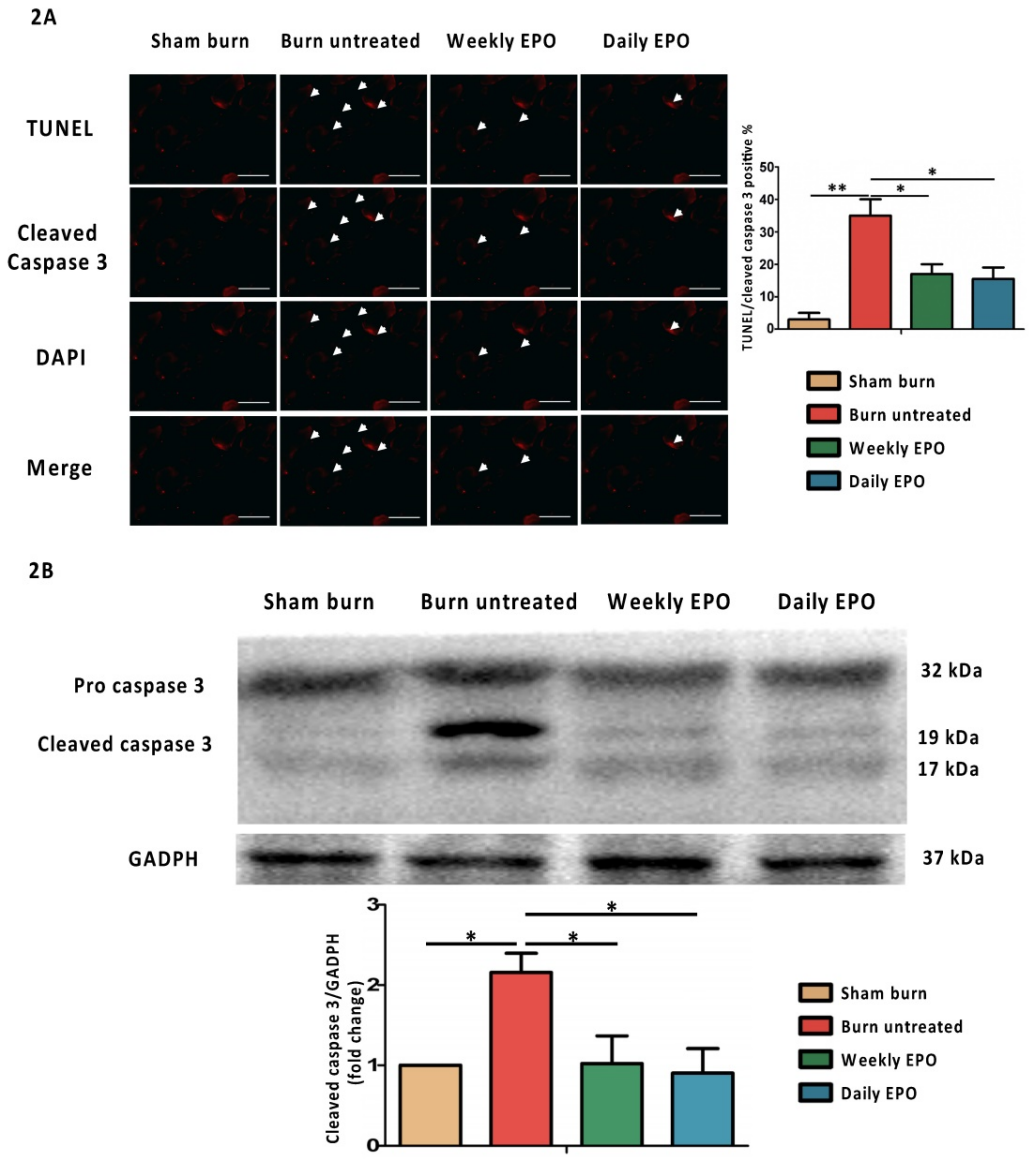
EPO on cleaved caspase 3 mediated apoptosis. (A) Representative TUNEL stain (green) and immunofluorescence of cleaved caspase 3 (red). DAPI (blue) was used for nuclear counterstaining. Arrowheads indicated positive-staining cells. EPO-treated groups showed less TUNEL/cleaved caspase 3 positive cells. (B) The expression of cleaved caspase 3 by western blot. EPO decreased the elevation of cleaved caspase 3 post-burn. All error bars represented the SEM. *p<0.05, **p<0.01. Scale bar: 50 µm.

For immunostaining localized to cleaved caspase 3, the nuclei for DAPI and TUNEL assay are shown in Figure 2A. The untreated burn group had an increased number of TUNEL-stained cells and the expression of cleaved caspase 3 (indicated by arrowhead). Both EPO-treated groups improved the phenomenon. The ratio of TUNEL/cleaved caspase 3 positive apoptotic cells was counted. We found an attenuation of the TUNEL/cleaved caspase 3 positive apoptotic cell ratio in both EPO-treated groups. Figure 2B shows the protein expression of cleaved caspase 3 by western blot. Cleaved caspase 3 was significantly decreased in both EPO-treated groups compared to the untreated burn group. We suggest EPO could attenuate caspase 3-mediated apoptosis post-burn.

### EPO attenuated apoptosis-inducing factor mediated apoptosis post-burn

**Figure 3:**
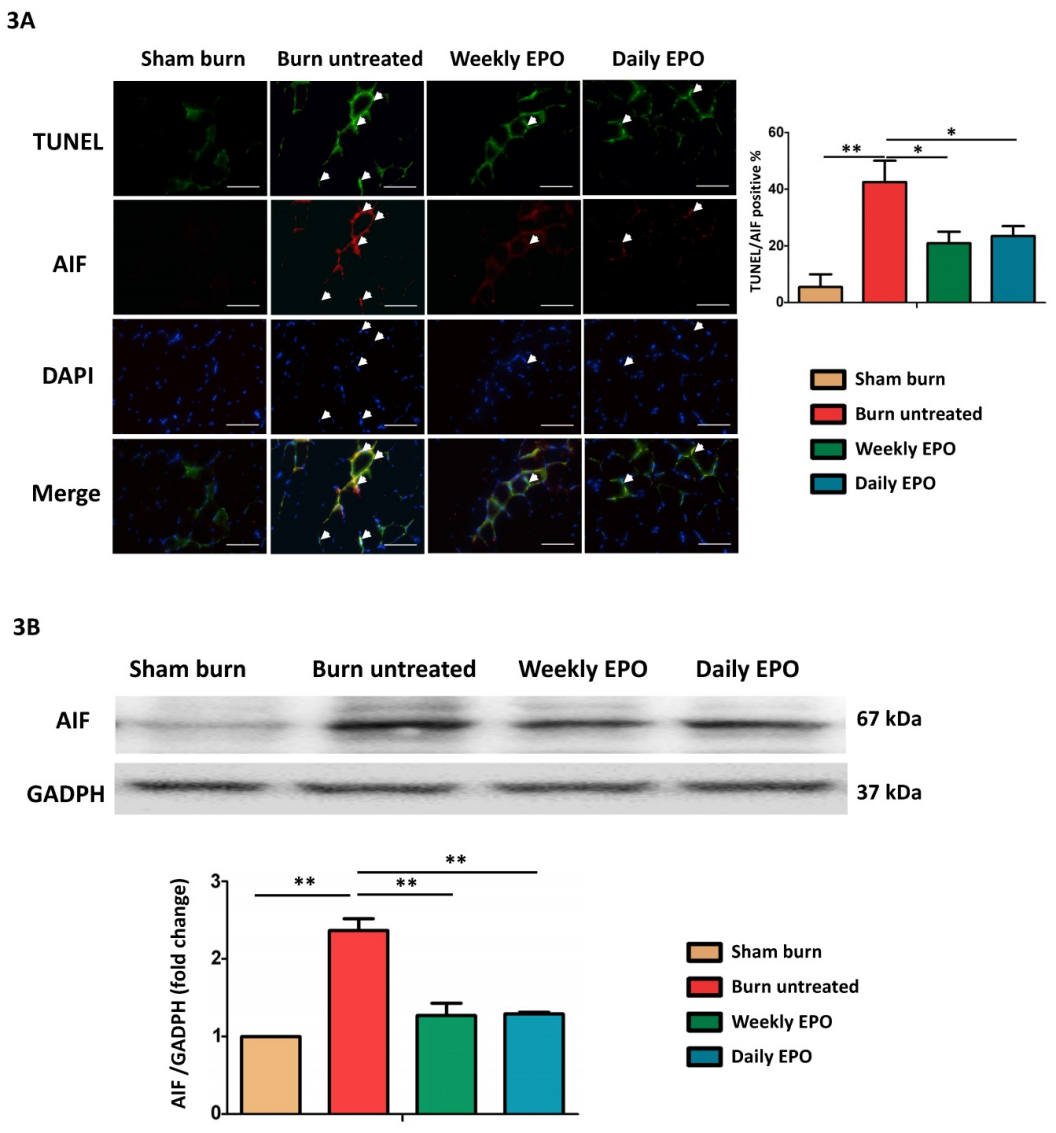
EPO on AIF mediated apoptosis. (A) Representative TUNEL stain (green) and immunofluorescence of AIF (red). DAPI (blue) was used for nuclear counterstaining. Arrowheads indicated positive-staining cells. TUNEL/AIF-positive cells were decreased in both EPO-treated groups. (B) Representative western blot of AIF. EPO decreased the elevation of AIF post-burn. AIF: Apoptosis-inducing factor. All error bars represented the SEM. *p<0.05, **p<0.01. Scale bar: 50 µm.

Caspase-independent pathway, apoptosis-inducing factor (AIF) might also be involved in apoptotic events post-burn. As shown in the merged image in Figure 3A, we found an increase in co-localized AIF with TUNEL-stained apoptotic cells (indicated by arrowheads) and EPO attenuated the phenomenon. EPO-treated groups had less TUNEL/AIF positive apoptotic cells. Figure 3B showed the western blot of AIF. The protein expression of AIF was elevated in untreated burn rats, but not in EPO-treated rats.

### EPO attenuated burn-induced extracellular matrix proteins overproduction

**Figure 4:**
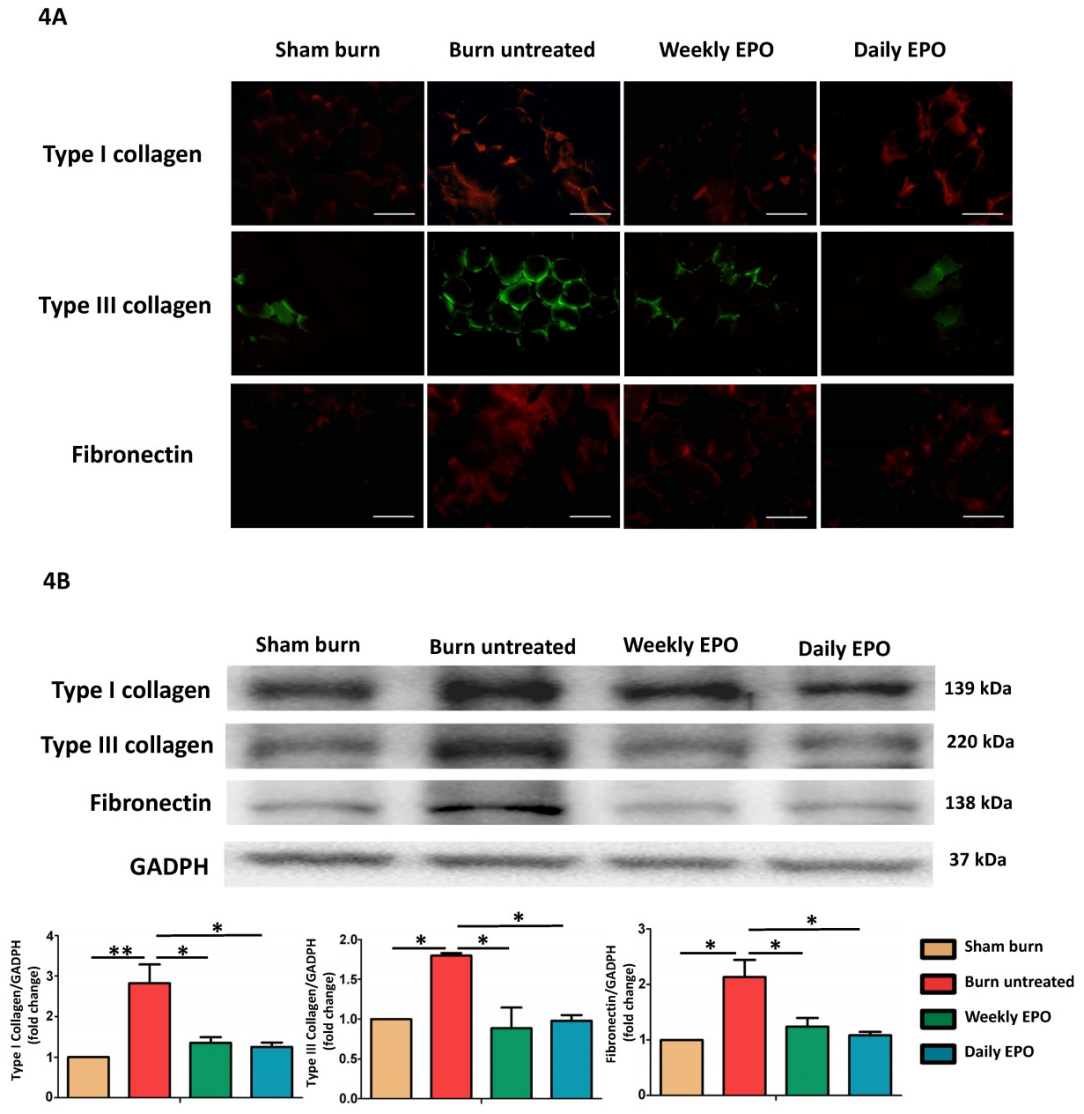
EPO on ECM overproduction following burn. (A) Immunofluorescence images of type I collagen, type III collagen and fibronectin. The expression of ECM proteins was significantly increased in untreated burn group but not in EPO-treated groups. (B) Western blot revealed EPO attenuated burn-induced ECM proteins elevation. ECM: extracellular matrix. All error bars represent the SEM. *p<0.05. Scare bars= 100 µm.

Figure 4A shows the immunofluorescence of ECM proteins (type I collagen, type III collagen, and fibronectin). The untreated burn rats had an overexpression of ECM proteins compared with other groups. Western blot in Figure 4B also showed that EPO attenuated ECM protein production following burn.

### EPO improved burn-induced muscle fibrosis by TGF-β1/Smad signaling to decrease CTGF expression

**Figure 5:**
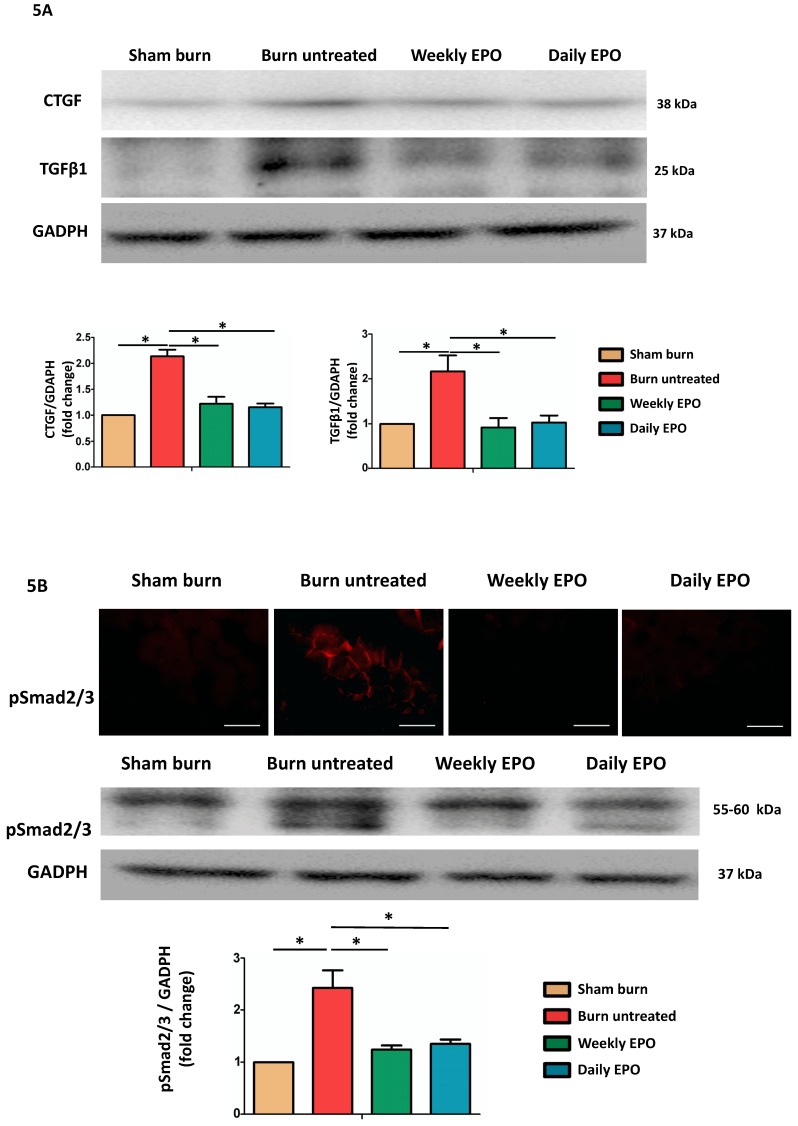
EPO alleviated burn-induced muscle fibrosis by suppressing TGF-β1/Smad pathway. (A) Representative western blot of CTGF and TGF-β1. EPO attenuated the overexpression of CTGF and TGF-β1 post-burn. (B) Immunofluorescence and western blot of pSmad2/3 in muscle sections. EPO attenuated the overexpression of pSmad2/3 after burn. TGF-β1: Transforming growth factor-β1, CTGF: Connective tissue growth factor, pSmad2/3: phosphorylated Smad2/3. *p<0.05.

Western blot was used to analyze the pro-fibrotic activity of TGF-β1 and CTGF expression (Figure 5A). Compared to sham-burn group, the untreated burn rats had a 2.7-fold and 2.1-fold increase in TGF-β1 and CTGF respectively. The EPO-treated groups attenuated their elevation post-burn. Furthermore, we found EPO decreased Smad 2/3 phosphorylation (pSmad2/3) by immunofluorescence and western blot (Figure 5B). We suggested EPO suppressed TGF-β1/Smad pathway to down-regulate CTGF expression post-burn.

## Discussion

Typically, critical illness including burn leads to muscle wasting for a prolonged period. In this study, we investigated gastrocnemius muscle at four weeks post-burn and the therapeutic potential of EPO in muscle wasting. We found EPO could prevent cleaved caspase-3 and AIF mediated apoptotic cell death as well as maintain muscle fiber diameter following burn. EPO also attenuated burn-induced collagens and fibronectin deposition. The possible mechanism mediated via TGF-β1/Smad2/3 signaling to decrease the expression of CTGF. EPO therapy attenuated burn-induced skeletal muscle wasting through anti-apoptotic and anti-fibrotic effects. Weekly EPO was non-inferior efficacy to daily EPO and did not alter red blood cell count. This study may provide information not only for burn patients but also possibly for those suffering from critical disease-induced muscle wasting.

Skeletal muscle wasting following critical illness is an important clinical feature and often associated with poor outcomes 6, 8, 72. To understand the underlying mechanisms and seek effective regimen to enhance patients' recovery are needed. Several mechanisms may contribute to burn-induced muscle wasting. Alteration of mitochondrial function 72-74 and activation of inflammatory cascades 1, 75 within skeletal muscle have been proposed. A marked increase of apoptotic cell death in skeletal muscle was found on day 7 post-burn 19 and caspase 3-mediated apoptosis has been implicated in various muscle atrophy models, including burn injury 20, 76, 77. Our data showed persistent caspase 3-mediated cell apoptosis in gastrocnemius muscle at four weeks post-burn to impend full recovery of muscle mass.

Prior to our study, the role of caspase-independent pathway in burn-induced muscle wasting was limited. Caspase-independent mechanisms, such as AIF lead to DNA fragmentation and cell death 25, 78-80. In this study, we found AIF expression was correlated with an increase of cell death and suggested caspase-independent apoptosis also contributed to burn-induced muscle atrophy.

Furthermore, muscle fibrosis is a progressive irreversible condition indicating impaired muscle healing process and delineated regeneration in response to aging, pathological disorders or trauma 27. Proper ECM remodeling process is important in muscle regeneration and the recovery of muscle strength 81. TGF-β is a key regulator to drive tissue atrophy and fibrosis 82, 83. Ex vivo TGF-β treated muscle experiment showed TGF-β induced mice muscle atrophy and reduced muscle contractility 84. TGF-β1 is the most abundant isoform of TGF-β in mammals. Mice with overexpressing TGF-β1 showed myofiber atrophy and collagen accumulation 85. During muscle damage, excessive TGF-β1 impairs ECM remodeling and initiates fibrotic cascade during the recovery phase 86. Targeting TGF-β1 could prevent ECM deposition and improve muscle regeneration in disuse muscle atrophy 87, toxin-induced muscle injury 88 as well as dystrophic and age-related muscle wasting 89. Smad2/3 and CTGF are the downstream mediators associated with TGF-β-induced fibrosis 90-93. Therapeutic agents inhibiting pro-fibrotic TGF-β1/Smad2/3 signaling could prevent renal fibrosis 94, liver fibrosis 95, 96, pulmonary fibrosis 97, 98, and cardiac fibrosis 99-101. This study showed burn enhanced the expression of TGF-β1, pSmad2/3 and CTGF to stimulate ECM (collagen and fibronectin) accumulation.

Erythropoietin is a multi-function cytokine that can regulate erythropoiesis and protect organs from damage. In an acute heart failure rat model, single (5,000 or 10,000 IU/kg) EPO injection decreased caspase-3 expression in renal medulla 102. Puchulu *et al*. suggested 3 days of EPO treatment (1,000 IU/kg) decreased oxidative damage and improved heart function in a rat hypovolemic model. Single-dose EPO (5,000 IU/kg) protected myocardial apoptosis in rats following carbon monoxide exposure 103. Overexpressed EPO improved rat heart fibrosis through the PI3K/Akt/TLR4 pathway to suppress the release of mediators such as TGF-β1, pro-inflammatory cytokines, and matrix metalloproteinase 58. EPO exerts neuroprotective effect in neurodegenerative diseases 104, ischemic brain injury 105, 106, spinal cord injury 107 and motor neuron death post-burn 69. EPO has also been found to improve muscular dysfunction in various experimental models, possible through increasing autophagy as well as decreasing apoptosis in type 2 diabetic skeletal muscles 65, reducing apoptosis in a crushing injury model 108 and preventing cancer-induced muscle alteration 67. A C2C12 myotube ischemia model also showed EPO and its derivations could decrease myotube cell death and inflammation 109. In human trials, weekly EPO injection increased mitochondrial capacity in skeletal muscle 110.

**Figure 6:**
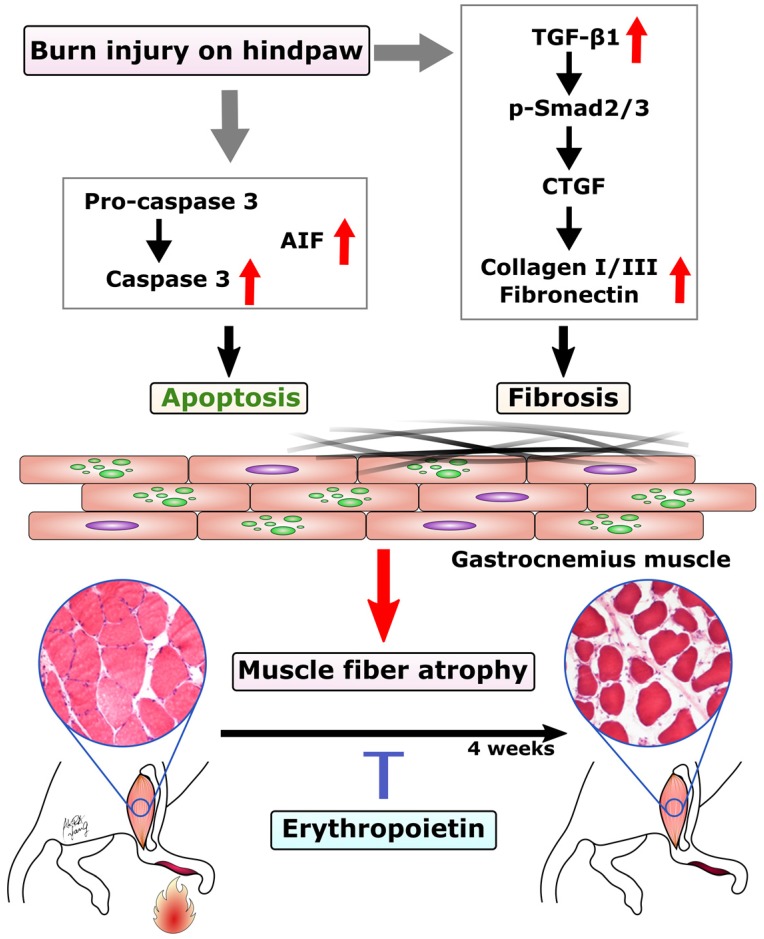
Proposed mechanism of EPO on muscle fiber atrophy following burn. EPO attenuates burn-induced skeletal muscle apoptosis via decreasing the expression of cleaved caspase 3 and AIF at four weeks post-burn. Moreover, EPO modulates burn-induced overexpression of TGF-β1/Smad2/3 profibrotic pathway and decreases the elevation of CTGF. EPO is a potential therapeutic agent for burn-induced skeletal muscle wasting. Apoptosis-inducing factor (AIF), Transforming growth factor beta 1 (TGF-β1), phosphorylated Smad2/3 (pSmad2/3), Connective tissue growth factor (CTGF).

In this study, we found EPO could prevent burn-induced muscle apoptosis through caspase-dependent and caspase-independent apoptotic pathways. In addition, EPO inhibited the expression TGF-β1 and its downstream signaling to prevent excessive ECM at four weeks post-burn. The possible mechanism of EPO on burn-induced muscle fiber atrophy was summarized in Figure 6. These finding suggests that EPO could potentially be used to preserve functional skeletal muscle tissue and attenuate excessive fibrotic tissue in burn patients.

## Conclusion

EPO attenuates burn-induced muscle wasting at 4 weeks post-burn. EPO preserves muscle fiber size and prevents apoptosis after burn through caspase-dependent as well as caspase-independent pathway. EPO improves excessive ECM production by suppressing TGF-β1/Smad signaling and CTGF overexpression following burn.

## Figures and Tables

**Table 1:** Effect of EPO on erythrocytosis

	Sham burn	Burn injury	Weekly EPO	Daily EPO	*p*^B-A^	*p*^C-B^	*p*^D-B^
RBC (x10^6^/μL)	7.51±0.34	7.84±0.99	8.07±0.88	10.41±0.87	0.709	0.069	0.001**

Data are presented as the mean ± SEM. **: p < 0.01. p^B-A^: burn-untreated versus sham-burn group, p^C-B^: Weekly EPO group versus burn untreated group p^D-B^: Daily EPO group versus burn untreated group. RBC: Red blood cell.

**Table 2:** TUNEL index in gastrocnemius muscle

Grouping	Sham burn	Burn untreated	Weekly EPO	Daily EPO
TUNEL-positive cell/HPF (x400)	4.32±3.76	33.56±5.84	16.07±6.82	13.23±6.36

Data was presented as the mean±SEM.

## Abbreviations

EPO: Erythropoietin
H&E staining: Hematoxylin & Eosin staining
TUNEL assay: TdT dUTP nick-end labeling assay
DAPI: 4'6-diamidino-2phenylinodole
AIF: Apoptosis-inducing factor
TGF-β1: Transforming growth factor beta 1
CTGF: Connective tissue growth factor
